# Individual Resonant Frequencies at Low-Gamma Range and Cognitive Processing Speed

**DOI:** 10.3390/jpm11060453

**Published:** 2021-05-23

**Authors:** Vykinta Parciauskaite, Evaldas Pipinis, Aleksandras Voicikas, Jovana Bjekic, Mindaugas Potapovas, Vytautas Jurkuvenas, Inga Griskova-Bulanova

**Affiliations:** 1 Institute of Biosciences, Life Sciences Centre, Vilnius University, Sauletekio ave 7, LT-10257 Vilnius, Lithuania; vykinta.parciauskaite@stud.gmc.vu.lt (V.P.); evaldas.pipinis@gmc.vu.lt (E.P.); aleksandras.voicikas@gmc.vu.lt (A.V.); mindaugas.potapovas@gmail.com (M.P.); 2 Human Neuroscience Group, Institute for Medical Research, University of Belgrade, Dr Subotica 4, 11000 Belgrade, Serbia; jovana.bjekic@imi.bg.ac.rs; 3 Department of General Psychology, Vilnius University, Universiteto 9/1, LT-01513 Vilnius, Lithuania; vytautas.jurkuvenas@fsf.vu.lt

**Keywords:** individual resonant frequency, gamma, cognitive performance, auditory steady-state response (ASSR), envelope following response (EFR)

## Abstract

Brain electrophysiological activity within the low gamma frequencies (30–80 Hz) has been proposed to reflect information encoding and transfer processes. The 40-Hz auditory steady-state response (40-Hz ASSR) is frequently discussed in relation to changed cognitive processing in neuropsychiatric disorders. However, the relationship between ASSRs and cognitive functioning still remains unclear. Most of the studies assessed the single frequency ASSR, while the individual resonance frequency in the gamma range (30–60 Hz), also called individual gamma frequency (IGF), has received limited attention thus far. Nevertheless, IGF potentially might better reflect individual network characteristics than standardly utilized 40-Hz ASSRs. Here, we focused on the processing speed across different types of cognitive tasks and explored its relationship with responses at 40 Hz and at IGFs in an attempt to uncover how IGFs relate to certain aspects of cognitive functioning. We show that gamma activity is related to the performance speed on complex cognitive task tapping planning and problem solving, both when responses at 40 Hz and at IGFs were evaluated. With the individualized approach, the observed associations were found to be somewhat stronger, and the association seemed to primarily reflect individual differences in higher-order cognitive processing. These findings have important implications for the interpretation of gamma activity in neuropsychiatric disorders.

## 1. Introduction

Brain electrophysiological activity within the low-gamma frequencies (30–80 Hz) has been proposed to reflect information encoding and transfer processes [1]. The gamma range oscillations have been linked to variety of perceptual processes [2,3] and cognitive functions [4,5,6,7]. Moreover, the impaired cognitive/perceptual processes, as observed in neuropsychiatric disorders, are often reflected in disturbed electrophysiological responses within the 30–80 Hz range [8,9]. It has therefore been proposed that the efficiency of neuronal information transfer in activated brain networks in the gamma range underlies the individual differences in cognitive performance [10].

One of the methods to explore the individual differences in neural synchronization in the gamma range is the auditory steady-state response (ASSR). The ASSR is an electrophysiological response of the brain that synchronizes to the frequency and phase of rapid, periodic auditory stimuli. The auditory stimulation evokes the greatest magnitude when stimuli are presented within the gamma range, especially around 40 Hz, and the evoked frequency is highly related to the frequency of stimulation [11,12].

Although the impairment of 40-Hz ASSRs is frequently put forward to reflect changed cognitive processing in neuropsychiatric disorders, especially schizophrenia [13,14,15,16,17], the relationship between ASSRs and cognitive functioning still remains unclear. In our recent critical review [18], we systematically analyzed existing findings on the associations between gamma-range ASSRs and cognitive functions in patients with neuropsychiatric or developmental disorders and healthy subjects. The evidence of the relationship between cognitive performance and ASSRs in pathological functioning was found across different studies (e.g., see [13,14,15,16,17,19,20,21,22,23,24,25,26]). However, there is not enough evidence in the literature to support this relationship in healthy participants.

One of the reasons why these effects have not been more prominent may lie in the resonance phenomena in the human auditory cortex. Namely, most of the previous studies assessed the single frequency ASSR, while the individual resonance frequency in the gamma range (30–60 Hz), also called individual gamma frequency (IGF [27,28,29]), has been mostly unexplored. In this respect, IGF represents the frequency at which the brain responds strongest in comparison to other frequencies when stimulated [30]. In other words, the key to the relationship between ASSR and cognitive performance may not necessarily be in the response to a single frequency such as 40 Hz, but in the response to person-dominant frequency within the gamma range.

This idea is especially compelling if the differences in cognitive performance are viewed as a continuum from “normal” to pathological [31], or more specifically, *superior-average-suboptimal/pre-clinical-pathological continuum.* The pathological conditions that display pronounced cognitive deficits are frequently accompanied by altered 40-Hz ASSRs [13]; however, the impairment is not limited to 40 Hz [21,24]. As the preferred oscillation frequencies of the networks are determined by the anatomical properties and the speed of neuronal communication [32], IGF potentially might better reflect individual network characteristics than standardly utilized 40-Hz ASSRs.

The responses at IGFs can be detected when multiple stimulation frequencies in the range under the interest are tested [30]. Alternatively, envelope following responses (EFRs, standing for a steady-state evoked response that follows the envelope of a stimulating waveform [33] to chirp stimulation covering the wide frequency window in one sweep [34]) show peak in the same frequency range as the individual preferred frequencies [34,35,36]. Although responses at IGFs and its individual variability have received limited attention thus far, recent research has provided evidence that frequency variation within the gamma range is related to certain functional aspects on the individual level in healthy subjects (i.e., the ability to detect small and sudden change in sound stimuli), [27,28,34] as well as in neuropsychiatric conditions (levels of psychopathology) [35,37].

However, the way responses at IGFs relate to certain aspects of cognitive functioning is not yet understood. Initial data on the gamma-range ASSRs in healthy participants showed that responses might be related to cognitive flexibility and reasoning (Tower of London task [38], Similarities [39], and the Mazes test [25]), as well as to behavioral indicators of processing speed (trial making test [25] and symbol coding [24]). Moreover, several studies showed a positive relationship between performance on gap detection task and the IGF in response to periodic stimulation [27,34]. Similarly, the individual resonant frequencies within the gamma range were negatively related to the speed on attentional control and executive tasks in patients with multiple sclerosis [19]. This suggests that the state of neural networks defining IGFs may reflect the temporal resolution, and thus may be related to the individual parameters of information processing speed. The information processing speed represents a fundamental capacity of the nervous system [40] that underlies higher-order cognitive functions such as learning, memory, and verbal and executive functions [41,42], and is often impaired in patients with neuropsychiatric and developmental disorders [43,44,45,46].

This study follows up on the idea that individual differences in the dominant gamma-band frequency may underlie the variability in the fundamental properties of cognitive functioning, even in the healthy population. Specifically, we focused on the processing speed across different types of cognitive tasks and explored its relationship with responses at 40 Hz and at IGFs. 

## 2. Methods

### 2.1. Subjects

Thirty-seven healthy right-handed subjects (17 females) without reported history of psychiatric and neurological disorders participated in the study (mean age ± SD 23.8 ± 4.7). The hearing thresholds of all subjects were within the normal range (<25dB HL at octave frequencies). Subjects abstained from alcohol for 24 h prior to the testing and did not consume nicotine and caffeine-containing drinks at least one hour prior to the experiment. The study was approved by the Vilnius Regional Biomedical Research Ethics Committee (no. 2020/3-1213-701), and all participants gave their written informed consent.

### 2.2. Procedure

The study was conducted in two blocks—cognitive assessment block in which participants performed a computerized battery of cognitive tasks, and EEG recording block, in which participants were exposed to auditory stimulation while the EEG was recorded. Cognitive assessment always followed EEG recordings.

### 2.3. Cognitive Assessment

The cognitive testing was performed using the Psychology Experiment Building Language [47]-based task battery, consisting of:

1. Simple reaction time task, in which participants were as asked to detect the presence of a visual stimulus (A letter) as quickly and accurately as possible.
2. Two-choice response reaction time task, in which participants had to indicate the direction of the displayed arrow by pressing the left or right button on a keyboard.
3. Lexical decision task, in which participants were asked to indicate if the correct word was presented or it contained a mistake.
4. Arithmetic decision task, in which subjects were presented with simple arithmetic expressions (simple additions or subtractions) and were asked to indicate whether the displayed outcome was correct or incorrect.
5. Semantic categorization task, in which the participants were successively presented with words, and their task was to indicate if the word belonged to the specific category, e.g., furniture, animal, utensils, etc.
6. Object judgement task assessing the mental rotation speed by making a decision as to whether two presented abstract waveforms are identical or different.
7. Tower of London task (ToL), generally considering tapping at planning and execution speed. In this task, participants had to move the colored disks to achieve the goal configuration in as few moves as possible.

As in this study we focused on processing speed, we therefore used response times (RTs) as the outcome measures across all tasks.

### 2.4. Auditory Stimulation

**Figure 1:**
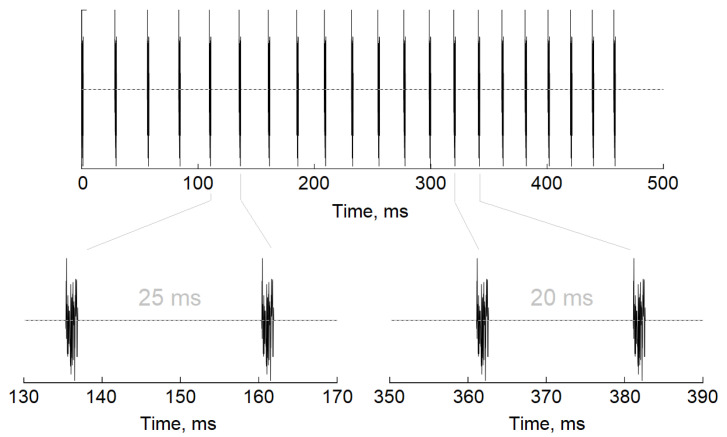
A schematic representation of chirp stimulus used in the study.

Click-based chirps consisting of 22 white noise bursts spaced with changing inter-click periods to cover 35–55 Hz range in 1 Hz step were used for the auditory stimulation. Duration of the inter-click period corresponded to the stimulation frequency (e.g., for 40 Hz stimulation, inter-click period was 25 ms for 50 Hz-20 ms; Figure 1). The chirp stimulation train lasted 475.4 ms. The auditory stimuli were designed in the Matlab 2014 environment (The MathWorks, Inc., Natick, MA, USA) and presented binaurally through Sennheiser HD 280 PRO earphones with sound pressure level adjusted to 60 dB with a DVM 401 dB meter (Velleman, TX, USA).

A total of 300 trains of chirps interspersed with single-frequency stimulation (not analyzed in the current work) were delivered with inter-stimulus intervals randomly set at 700–1000 ms. Subjects were asked to focus on stimulation and mentally count randomly presented single clicks interspersed within periodic sounds and to report the presented number after each of stimulation run in order to keep subjects’ attention towards stimulation.

### 2.5. EEG Recording

EEG was recorded with an ANT device (ANT Neuro, Hengelo, the Netherlands) and a 64 channel WaveGuard EEG cap (International 10–20 System) with Ag/AgCl electrodes. Mastoids were used as a reference; the ground electrode was attached close to Fz. Impedance was kept below 20 kΩ, and the sampling rate was set at 1024 Hz. Simultaneously, vertical and horizontal electro-occulograms (VEOG and HEOG) were recorded from above and below the left eye and from the right and left outer canthi.

### 2.6. EEG Processing

The off-line pre-processing of EEG data was performed in EEGLAB for MatLab© 2014 [48,49]. The power-line noise was removed using multi-tapering and Thomas F-statistics, as implemented in CleanLine plugin for EEGLAB. Data were visually inspected, and channels with substantial noise (shift, movements) throughout the recording were manually rejected. An independent component analysis (ICA) was performed on the remaining channels with the ICA-implementation of EEGLAB (“runica” with default settings), and independent components related to eye movements were removed.

The further data analysis was based on the usage of custom written scripts based on EEGLAB [48] and Fieldtrip functions [50]. Epochs were created from −500 ms to 1100 ms post-stimulus onset. Data were baseline-corrected to the mean of the pre-stimulus period, and epochs were further visually inspected for the remaining artefacts. A wavelet transformation was performed utilizing complex Morlet wavelet from Matlab© Wavelet Toolbox with frequencies represented from 1 to 120 Hz, with 1 Hz intervals between each frequency.

The phase-locking index (PLI), corresponding to the phase consistency over the trials, and the event-related spectral perturbation (ERSP), indicating event-related changes in power relative to a pre-stimulus baseline, were calculated according to the following formulas [49]:

(1) $$PLI\left(c,f,t\right)=\frac{1}{N}\sum_{n}^{N}\frac{X\left(c,f,t,n\right)}{\left|X\left(c,f,t,n\right)\right|},$$

(2) $$ERSP\left(c,f,t\right)=\frac{1}{N}\overset{N}{\underset{n}{\sum }}|X(c,f,t,n{|}^{2},$$

where for every channel *c*, frequency *f*, and time point *t*, a measure is calculated by taking time frequency decomposition *X* of each trial *n*.

**Figure 2:**
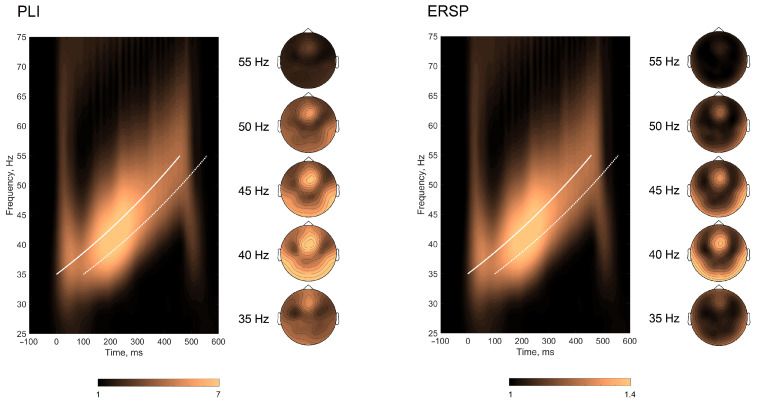
Time-frequency plots of PLIs and ERSPs for envelope following response. The white solid line corresponds to the course of auditory stimulation; the white dashed line denotes +100 ms window from the stimulation line. The grand-averaged topographies for envelope-following response at 35, 40, 45, 50, and 55 Hz stimulation are presented alongside the time-frequency plots.

For the baseline correction, the signal during stimulation was divided by the signal averaged from −400 to 0 ms for each frequency. The topographical representation of the response resembled classical distribution observed for auditory-evoked gamma-range responses with a clear fronto-central distribution [38]; thus, the extracted PLIs and ERSPs at frequencies spanning 35–55 Hz were grouped for the fronto-central (Fz, Cz, FCz, C1, C2, F1, F2, FC1, FC2) region where responses were most pronounced (Figure 2).

For the responses to chirps, the curve representing time-frequency points of the stimulation (starting with the first click presented) was used to define the exact time points for each stimulation frequency (seen as a white bold line in the time-frequency plot in Figure 2). The average response to each stimulation frequency (from 35 to 55 Hz in 1 Hz steps) was calculated using a time window of +100 ms from the stimulation line (consistently with observed response windows in the time-frequency plots, seen as a white dashed line in Figure 2). The following measures were extracted: PLI/ERSP values at 40 Hz (40 Hz EFR), and PLI/ERSP values at the maximal response (further referred to as IGF-EFR).

### 2.7. Statistical Analysis

Descriptive statistics (means and standard deviations) were calculated for all variables in the study. PLI and ERSP values for 40 Hz and IGF were compared using paired sample *t*-test. For cognitive tasks, we performed principal component analysis to extract common latent dimension and assess the individual task loading. Pearson‘s correlation coefficients were calculated to assess the relationship between RTs from cognitive tasks and PLI/ERSP measures at 40 Hz, as well as at IGFs. To account for multiple comparisons, we Bonferroni-corrected the threshold for statistical significance, and the *p*-values less than 0.004 (0.05/13) were regarded as significant. Statistical analysis was performed using SPSSv20 (SPSS Inc., Chicago, IL, USA). In addition, we used JASP (version 0.14.1) [51] to conduct Bayesian analysis. To provide additional information on the level of evidence, we report Bayesian factors and credibility intervals for correlations between measures of cognitive processing speed and EEG measures.

## 3. Results

### 3.1. Cognitive Performance

The means and standard deviations of RTs on cognitive tasks are presented in Table 1. To assess the latent structure and verify common source of variance across all tasks, we conducted principal component analysis.

As expected, the first principal component accounted for almost half of the variance of RTs across different tasks (46.4%); however, two additional components with eigenvalues >1 emerged. Table 1 shows loadings of RTs for each task on all three components. Notably, all cognitive tasks except ToL showed primary loading on the first principal component. This indicates that RTs on ToL had a significant amount of variance that was task-specific, i.e., which was not shared with other cognitive speed tasks.

### 3.2. Envelope Following Responses

**Figure 3:**
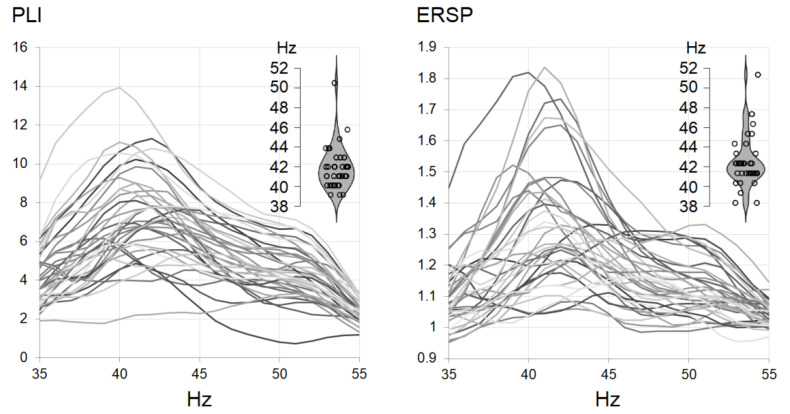
Individual phase-locking index (**PLI**) and event-related spectral perturbation (**ERSP**) curves with individual gamma frequency distributions.

The grand averaged time-frequency representation of PLIs and ERSPs is plotted in Figure 2, along with the topographical plots of EFRs at 35, 40, 45, 50, and 55 Hz. EFRs resembled classical fronto-central topographies. The extracted individual PLI and ERSP curves are plotted in Figure 3. IGFs spanned the frequency range of 36–53 Hz with mean maximums observed around 41–42 Hz. The PLI and ERSP values were extracted at 40 Hz and at IGFs. The means and standard deviations of PLIs, ERSPs, and IGFs are presented in Table 2.

### 3.3. The Relationship between EEG Measures and Cognitive Processing Speed

**Figure 4:**
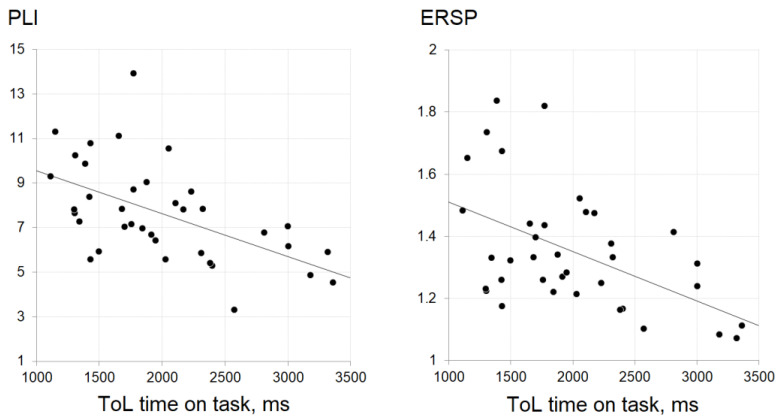
Scatterplots of PLIs and ERSPs at IGFs against the Tower of London task response times.

To explore the relationship between EFRs and cognitive functions, we first calculated the Pearson’s correlation coefficients for all measures separately (Table 3). RTs from the ToL task showed significant associations to EEG measures: negative correlations were observed between RTs on ToL and PLIs, as well as ERSPs for responses at both 40 Hz and at IGFs. Of note, the correlations with cognitive speed measures at IGFs were very similar to those at 40 Hz, which was to be expected as both PLI and ERSP were highly correlated (r > 0.95); still, the associations appeared to be somewhat stronger for responses at IGFs (PLI: 40-Hz EFR BF_10_ = 23.19 vs. IGF-EFR BF_10_ = 81.78; ERSP: 40-Hz EFR BF_10_ = 21.10 vs. IGF-EFR BF_10_ = 30.40). Scatterplots of PLIs and ERSPs at IGFs against mean move times in the Tower of London task are presented in Figure 4. No correlations were observed for RTs on other tasks. The Bayesian factors and credibility intervals for all correlations are provided in the Supplementary Material.

To understand the nature of this association, we assessed whether EFR measures were related to general or unique processing speed variance form ToL. Namely, we correlated EFR measures with (a) first principal component (i.e., global processing speed variance that is shared across all cognitive tasks) and (b) unique variance of ToL (i.e., the residual variance of ToL when the global processing speed of other cognitive tasks is regressed out). The results showed zero correlations with the latent factor of global processing speed but showed stable correlations with ToL unique variance at 40 Hz (r_PLI_ = −0.495, *p* = 0.002; r_ERSP_ = −0.491, *p* = 0.002) as well as at IGFs (r_PLI_ = −0.537, *p* = 0.001; r_ERSP_ = −0.483, *p* = 0.002). Again, the correlation for PLI at IGFs was slightly higher than for 40 Hz when observed under the Bayesian model (PLI: 40-Hz EFR BF_10_ = 13.96 vs. IGF-EFR BF_10_ = 30.09; ERSP: 40-Hz EFR BF_10_ = 20.03 vs. IGF-EFR BF_10_ = 19.82). On the basis of the classification scheme for interpreting Bayesian factors [52], PLI at 40 Hz provides strong evidence, while the PLI at IGFs provides very strong evidence towards the hypothesis.

## 4. Discussion

ASSRs in the gamma range are frequently regarded as an index of impaired cognitive functioning in patients with schizophrenia [13,16,17]. Nevertheless, the association between ASSRs and cognitive domains is not that clear [18]. The 40-Hz ASSRs have received the most attention both from the clinical perspective [13,16,17,53] and as individual markers of the ability to generate synchronous gamma activity [54,55]. However, the individual resonant frequency phenomenon was observed [30], suggesting that responses assessed at IGFs might better reflect individual differences and more robustly translate into certain cognitive performance patterns.

We obtained the envelope following responses to click trains spaced in a logarithmic manner similar to chirps covering the frequency range within 35–55 Hz. IGFs were estimated as the stimulation frequencies producing the strongest and most synchronized responses [30]. A group mean maximum was observed at around 41–42 Hz with the individual peaks estimated within the 35–53 Hz range, being in line with previous reports [27,28,30,56]. We extracted phase-locking index and event-related spectral perturbation at 40 Hz (40-Hz EFR) and at IGFs (IGF-EFR) in order to be able to compare results to the existing 40-Hz ASSR literature. Both PLIs and ERSPs obtained at 40 Hz and IGFs were highly correlated, resembling topographical distribution corresponding to classical ASSRs [38,57] with a clear fronto-central locus (Figure 2). Although we did not assess 40-Hz ASSR, we believe that EFRs at 40 Hz capture similar brain activity, as can be seen from the topographical activation pattern.

We evaluated processing speed on different cognitive tasks that reveal simple and complex information processing, the later covering semantic, spatial, arithmetic, and lexical aspects. However, the only observed association to gamma-range responses was a negative correlation between mean response times on ToL task and measures at both 40 Hz and at IGFs. Interestingly, the results showed that the observed relationship was unique to the ToL speed variance. This negative association between response times and the level of synchronization showed that subjects with better synchronization properties in this study were faster when a planning/problem solving task was performed. This finding is in line with sparse earlier observations showing a positive correlation between the performance on the complex planning and reasoning tasks such as the Mazes test from MATRICS and Similarities and the phase-locking properties of 40-Hz ASSR in both patients with schizophrenia and controls [24,25], indicating better performance in subjects with more synchronized ASSRs. Additionally, gamma-range ASSRs in healthy subjects were positively related to behavioral indicators of processing speed on two other multifaceted tests—trial making test [25] and symbol coding [24]. In our previous work, we did not observe an association between parameters of 40-Hz ASSRs and response times on a set of cognitive tasks. However, we observed a positive association between parameters of 40-Hz ASSR and number of moves made on the Tower of London task [38]. Taken together, the relationship to measures of gamma activity suggests that synchronization properties of the brain shape individual potential to perform complex reasoning and planning tasks. Moreover, this type of response does not reflect simple motor or sensory processes but rather higher-order speed of cognitive performance. To this point, it was previously suggested that individual performance of ToL task depends on elaboration of diverse strategies [58], with different working memory [59,60] and abstract thinking [61,62] demands and distinct brain activation patterns [63]. Moreover, ToL is sensitive to trait variance in levels of impulsivity [60]. Thus, the finding that the lower number of moves in ToL [38] and higher response speed (current study) are related to higher synchronization level implies that these neurophysiological processes underline distinct cognitive subdomains.

In line with our expectations, responses at IGFs showed somewhat stronger association to behavioral measures than responses at 40 Hz. This trend was even more prominent when ToL unique variance was assessed. The fact that both responses at 40 Hz and at IGFs were related to the same cognitive domain measure was expected. We have previously shown that both 40-Hz ASSRs and responses to chirp stimulation within 38–43 Hz were related to clinical assessment scores in patients with disorders of consciousness [64,65]. Although the individual peaks estimated in this study were within the 35–53 Hz range, corresponding to the ranges reported in previous reports [27,28,30,56], the majority of subjects had their IGFs at 40–42 Hz. Gransier et al. suggested that the scalp-recorded ASSRs within the 30–60 Hz range originate from the same generators and the choice of stimulation frequency does not have a large effect on relative measures, even though peak frequencies differ across subjects [56]. This assumption is supported by similar topographies observed at different frequencies, as can be seen in Figure 2 and by our recent studies in clinical populations utilizing chirp stimulation where the significant associations with clinical symptoms spanned a certain frequency range: hallucination scores and PLIs in patients with schizophrenia were associated within the frequency range of 32–43 Hz [35], and a correlation between Coma Rating Scale—Revised total score in patients with disorder of consciousness was detected within the 38–42 Hz window [64,65]. However, individual peak frequencies of gamma oscillations were shown to determine temporal resolution, i.e., reflect the individual ability to detect small and sudden change in sound stimuli [27,28]. In patients with multiple sclerosis, IGFs were negatively related to the speed on attentional control and executive tasks [19]. We expected that the state of neural networks defining individual gamma frequencies would also reflect the temporal dynamics on more general cognitive tasks reflecting different aspects of simple and complex information processing. However, as shown by the lack of association between IGFs and processing speed in our sample, it is possible that connection can be observed in clinical populations with clearly impaired cognitive processing, or be highly modality-specific (i.e., auditory response is associated to the performance of auditory tasks). The latter assumption connects with a recent suggestion of Molina et al. [66] that evoked gamma may be an index of the brain’s overall “adaptive integrity“ of the lower-level perceptual networks. We tested healthy young participants, and all tasks utilized in the current research were based on visual domain assessment. Future research should combine visual and auditory domain-based processing in order to untangle the role that task modality plays in this relationship. Finally, it should be acknowledged that the sample size is not sufficient to reliably detect small-to-medium-sized correlations. Therefore, strength of evidence for the lack of the relationship between processing speed across different tasks and gamma oscillations needs to be replicated in a larger sample.

## 5. Conclusions

Gamma activity, as a response at 40 Hz and at IGFs, is related to the performance speed on complex cognitive task tapping planning and problem solving. With the individualized approach, the observed associations is somewhat stronger, and the association seem to primary reflect individual differences in higher-order cognitive processing. These findings are particularly important for the interpretation of gamma activity in neuropsychiatric disorders.

## Figures and Tables

**Table 1:** **Means, standard deviations and** loadings of each task RT on all three components.

Task	RT (ms)		Principal Component Loadings		
	Mean	SD	Component 1	Component 2	Component 3
Simple reaction time task	294.38	50.75	0.65 *	0.59	−0.33
Two-choice response time task	378.16	61.19	0.76 *	0.51	-
Arithmetic decision task	1116.12	327.84	0.72 *	-	0.31
Lexical decision task	1248.64	357.79	0.63 *	−0.62	−0.40
Semantic categorization task	751.97	209.61	0.84 *	−0.44	-
Object judgement task	814.68	188.15	0.61 *	-	0.41
Tower of London task	1997.52	626.12	0.51	-	0.62 *

Note: For PCA the loadings <0.30 were suppressed; primary loadings marked with *.

**Table 2:** Means and standard deviations of PLIs and ERSPs at 40 Hz and at IGFs.

		40-Hz EFR	IGF-EFR	*t*-Test	IGF
PLI	Mean	7.07	7.63	−6.534, *p* < 0.001	41.89
	SD	2.39	2.20		2.27
ERSP	Mean	1.29	1.35	−6.849, *p* < 0.001	42.19
	SD	0.20	0.20		2.57

EFR—envelope following response; IGF—individual gamma frequency; IGF-EFR—envelope following response at individual gamma frequency.

**Table 3:** Correlation coefficients and corresponding *p*-values for correlations between envelope following response measures and response times on cognitive tasks.

Task		PLI			ERSP		
		40-Hz EFR	IGF-EFR	IGF	40-Hz EFR	IGF-EFR	IGF
Simple reaction time task	r	0.05	0.04	−0.03	0.03	0.03	0.01
	*p*	0.79	0.83	0.86	0.86	0.85	0.94
Two-choice response time task	r	0.08	0.02	−0.18	0.09	0.03	−0.20
	*p*	0.62	0.93	0.29	0.60	0.87	0.23
Arithmetic decision task	r	−0.13	−0.15	−0.06	−0.12	−0.16	−0.12
	*p*	0.46	0.36	0.71	0.47	0.34	0.49
Lexical decision task	r	−0.10	−0.11	−0.02	−0.03	−0.07	−0.18
	*p*	0.58	0.52	0.90	0.87	0.70	0.28
Semantic categorization task	r	−0.20	−0.23	−0.04	−0.15	−0.18	−0.10
	*p*	0.23	0.18	0.80	0.39	0.28	0.55
Mental rotation task	r	−0.16	−0.16	0.21	−0.10	−0.12	0.10
	*p*	0.35	0.34	0.21	0.54	0.47	0.57
Tower of London task	r	*−0.50*	*−0.55*	0.08	*−0.49*	*−0.51*	0.09
	*p*	*0.002*	*<0.001*	0.65	*0.002*	*0.001*	0.60

EFR—envelope following response; IGF—individual gamma frequency; IGF-EFR—envelope following response at individual gamma frequency.

## Data Availability

The data presented in this study are available on request from the corresponding author. The data are not publicly available due to privacy restrictions.

## Supplementary Materials

The following are available online at https://www.mdpi.com/article/10.3390/jpm11060453/s1, Table S1: Bayesian correlation outcomes between EEG measures and cognitive measures.
