# Supplementary material for: Individual Resonant Frequencies at Low-Gamma Range and Cognitive Processing Speed
Source: J Pers Med. 2021 May 23;11(6):453. doi: 10.3390/jpm11060453 (PMC8224604; doi:10.3390/jpm11060453)
Supplement: Supplementary file 1 [file jpm-11-00453-s001.zip › jpm-1157936-supplementary.pdf]

**Table S1.** Bayesian correlation outcomes between EEG measures and cognitive measures.

Here we use the following terms to interprete the level of evidence: Antecdotal $BF_{10} > 3$ Moderate $BF_{10} > 3$; Strong - $BF_{10} > 10$; Very strong - $BF_{10} > 30$; Extreme - $BF_{10} > 100$

**Bayesian Pearson Correlations** 2-Choice response time task

| | PLI | | | ERSP | | |
|---|---|---|---|---|---|---|
| | **40-Hz EFR** | **IGF** | **IGF-EFR** | **40-Hz EFR** | **IGF** | **IGF-EFR** |
| Pearson's r | 0.084 | -0.180 | 0.015 | 0.089 | -0.202 | 0.028 |
| $BF_{10}$ | 0.230 | 0.354 | 0.205 | 0.233 | 0.409 | 0.207 |
| Upper 95% CI | 0.383 | 0.148 | 0.325 | 0.387 | 0.127 | 0.336 |
| Lower 95% CI | -0.237 | -0.461 | -0.299 | -0.233 | -0.478 | -0.287 |

**Bayesian Pearson Correlations** Simple reaction time task

| | PLI | | | ERSP | | |
|---|---|---|---|---|---|---|
| | **40-Hz EFR** | **IGF** | **IGF-EFR** | **40-Hz EFR** | **IGF** | **IGF-ERF** |
| Pearson's r | 0.046 | -0.030 | 0.036 | 0.031 | 0.013 | 0.031 |
| $BF_{10}$ | 0.212 | 0.208 | 0.209 | 0.208 | 0.205 | 0.208 |
| Upper 95% CI | 0.351 | 0.286 | 0.343 | 0.339 | 0.324 | 0.339 |
| Lower 95% CI | -0.272 | -0.338 | -0.280 | -0.285 | -0.300 | -0.285 |

**Bayesian Pearson Correlations** Arithmetic decision task

| | PLI | | | ERSP | | |
|---|---|---|---|---|---|---|
| | **40-Hz EFR** | **IGF** | **IGF-EFR** | **40-Hz EFR** | **IGF** | **IGF-ERF** |
| Pearson's r | -0.125 | -0.064 | -0.155 | -0.123 | -0.117 | -0.162 |
| $BF_{10}$ | 0.266 | 0.219 | 0.306 | 0.264 | 0.257 | 0.319 |
| Upper 95% CI | 0.200 | 0.256 | 0.172 | 0.202 | 0.208 | 0.165 |
| Lower 95% CI | -0.417 | -0.366 | -0.441 | -0.415 | -0.410 | -0.447 |

**Bayesian Pearson Correlations** Lexical decision task

| | PLI | | | ERSP | | |
|---|---|---|---|---|---|---|
| | **40-Hz EFR** | **IGF** | **IGF-EFR** | **40-Hz EFR** | **IGF** | **IGF-ERF** |
| Pearson's r | -0.095 | -0.021 | -0.109 | -0.027 | -0.183 | -0.066 |
| $BF_{10}$ | 0.238 | 0.206 | 0.250 | 0.207 | 0.360 | 0.220 |
| Upper 95% CI | 0.228 | 0.294 | 0.215 | 0.288 | 0.145 | 0.254 |
| Lower 95% CI | -0.392 | -0.330 | -0.404 | -0.335 | -0.463 | -0.368 |

**Bayesian Pearson Correlations** Semantic categorisation

| | PLI | | | ERSP | | |
|---|---|---|---|---|---|---|
| | **40-Hz EFR** | **IGF** | **IGF-EFR** | **40-Hz EFR** | **IGF** | **IGF-ERF** |
| Pearson's r | -0.203 | -0.042 | -0.227 | -0.145 | -0.101 | -0.183 |
| $BF_{10}$ | 0.412 | 0.211 | 0.492 | 0.292 | 0.243 | 0.361 |
| Upper 95% CI | 0.126 | 0.275 | 0.102 | 0.181 | 0.222 | 0.145 |
| Lower 95% CI | -0.479 | -0.348 | -0.497 | -0.433 | -0.397 | -0.463 |

**Bayesian Pearson Correlations** Object judgement task

| | PLI | | | ERSP | | |
|---|---|---|---|---|---|---|
| | **40-Hz EFR** | **IGF** | **IGF-EFR** | **40-Hz EFR** | **IGF** | **IGF-ERF** |
| Pearson's r | -0.158 | 0.213 | -0.163 | -0.103 | 0.095 | -0.122 |

**Bayesian Pearson Correlations** Object judgement task

| | PLI | | | ERSP | | |
|---|---|---|---|---|---|---|
| | **40-Hz EFR** | **IGF** | **IGF-EFR** | **40-Hz EFR** | **IGF** | **IGF-ERF** |
| $BF_{10}$ | 0.311 | 0.442 | 0.320 | 0.244 | 0.238 | 0.263 |
| Upper 95% CI | 0.169 | 0.486 | 0.164 | 0.220 | 0.392 | 0.203 |
| Lower 95% CI | -0.443 | -0.116 | -0.447 | -0.399 | -0.227 | -0.414 |

**Bayesian Pearson Correlations** Tower of London

| | PLI | | | ERSP | | |
|---|---|---|---|---|---|---|
| | **40-Hz EFR** | **IGF** | **IGF-EFR** | **40-Hz EFR** | **IGF** | **IGF-ERF** |
| Pearson's r | -0.498 * | 0.076 | -0.550 ** | -0.494 * | 0.088 | -0.510 ** |
| $BF_{10}$ | 23.192 | 0.226 | 81.778 | 21.100 | 0.233 | 30.395 |
| Upper 95% CI | -0.194 | 0.377 | -0.260 | -0.189 | 0.386 | -0.209 |
| Lower 95% CI | -0.693 | -0.245 | -0.729 | -0.691 | -0.234 | -0.702 |

* $BF_{10} > 10$, ** $BF_{10} > 30$, *** $BF_{10} > 100$
